# Optimized Digestion Conditions for Membrane Protein Footprinting and Mass Spectrometry Analysis

**DOI:** 10.3390/membranes16060215

**Published:** 2026-06-22

**Authors:** Ming Cheng, Xinzhu Li, Lin Bai, Weikai Li, Michael L. Gross

**Affiliations:** 1 Shandong Laboratory of Yantai Drug Discovery, Bohai Rim Advanced Research Institute for Drug Discovery, Yantai 264117, China; 2 Shanghai Institute of Materia Medica, Chinese Academy of Sciences, Shanghai 201203, China; 3 Department of Chemistry, Washington University, St. Louis, MO 63130, USA; li.xinzhu@email.wustl.edu; 4 Department of Biochemistry and Molecular Biophysics, Washington University, St. Louis, MO 63130, USA

**Keywords:** membrane protein, vitamin K epoxide reductase (VKOR), mass spectrometry, protein footprinting, filter-aided sample preparation (FASP), membrane protein digestion

## Abstract

Integral membrane proteins (IMPs), which constitute 50–60% of drug targets, play essential roles in numerous biological processes but remain underrepresented in conventional bottom-up and structural proteomics owing to their hydrophobicity and resistance to proteolysis. Although advances in IMP proteomics have improved global IMP detection, most efforts focus on proteome-scale protein identification rather than targeted structural analysis. Protein footprinting and cross-linking, two approaches in structural proteomics, require high sequence coverage and protein digestion to peptides of suitable length for structural elucidation, necessitating optimized digestion condition for individual IMPs. Here, we report a digestion protocol tailored for structural mass spectrometry and evaluate its performance by using a single amphipathic IMP model featuring distinct extramembrane and transmembrane domains. We evaluated the use of various protease–additive combinations and applied filter-aided sample preparation (FASP) to remove detergents and surfactants efficiently prior to MS analysis. The optimized conditions consistently yielded >90% sequence coverage. Guided by MS retention time calibration and hydrophobic factor simulations, we identified a “sweet spot” for transmembrane peptide detection. Notably, although cleavable surfactants can enhance proteome-wide coverage, our results show that they are not essential for single protein studies as they are in structural proteomics. Instead, detergent removal, protease selection, and generation of suitably sized peptides are critical for enabling reliable bottom-up structural analysis of IMPs. The protocol developed here provides a practical framework for optimizing digestion conditions in IMP characterization.

## 1. Introduction

Integral membrane proteins (IMPs) are involved in numerous biological and pharmacological functions, including intercellular communication, cellular development, signal transduction, cell migration, and drug resistance. Whereas IMPs make up ~30% of the human proteome, IMPs constitute nearly 60% of current drug targets [1]. Owing to their hydrophobic and dynamic properties, determining the structure of IMPs, especially those of eukaryotic origin, remains a challenge when using conventional methods [2]. Thus, advances in analytical technologies to improve characterization and to understand IMPs are highly desired.

Structural mass spectrometry (MS) (sometimes known as structural proteomics) is an emerging tool to characterize the high-order structure of proteins as well as their primary structure [3,4,5,6,7,8]. MS analysis for IMPs, however, poses a challenge due to their highly hydrophobic nature, resulting in poor solubility in aqueous buffer, low ionization efficiency, lack of tryptic residues, and poor sample recovery owing to precipitation and aggregation [9,10].

To overcome these challenges, various methods have been developed to improve the efficacy of IMP bottom-up analysis. The methods include new approaches in sample preparation (e.g., protein solubilization, separation, and digestion) as well as refinement of the MS analysis [11,12]. Specifically, chemical additives such as chaotropic reagents [13], surfactants [13,14,15,16,17,18], organic solvents [19], and organic acids [20] have been used to solubilize IMPs. Li and coworkers [21] described a microwave-assisted strategy to physically enhance IMP solubilization. For IMP separation and enrichment, density-gradient centrifugation [22], detergent-based extraction following centrifugation [23], glycan moiety-directed enrichment [24], and protein surface chemical labeling [25,26], were employed for enrichment of IMPs, and these methodsovercame some of the challenges of detecting low-abundance IMPs. To improve proteolytic efficiency, Hettich and coworkers [27] developed a multienzyme digestion strategy that uses sample filtration to recover undigested proteins for additional proteolytic digestion. These methods have greatly improved the coverage of IMPs.

Despite these effects and significant improvements in membrane proteomics, most proteomic studies aim to improve IMP detection on a proteome scale; that is, to increase the percentage or number of detected proteins and to improve the confidence of identification. There is, however, a notable absence of systematic studies of individual membrane proteins under several proteolytic conditions. Protein footprinting [28,29,30,31,32,33,34,35,36,37], hydrogen deuterium exchange (HDX) [38], and cross-linking typically focus on specific proteins or protein complexes, necessitating high sequence coverage to map the protein surface thoroughly and to acquire comprehensive structural information. Achieving a high level of coverage necessitates the optimization of enzymatic digestion to generate peptides with suitable length and sufficient abundance for MS analysis. Further, sample preparation conditions should be carefully optimized to maximize digestion efficiency without adversely affecting the protease activity or interfering with downstream MS analysis. For example, HDX-MS workflows require meticulous phospholipid removal to prevent LC-MS system contamination. This removal involves extensively screening digestion conditions to attain sufficient sequence coverage [39,40,41]. Stable covalent labeling-based IMP footprinting offers more resilience in downstream handling without sacrificing structural information. Progress includes the work of Li and coworkers [42], who developed a protocol to footprint IMPs in live cells, followed by in-gel protein purification and digestion. Wang and colleagues [43] employed multiple enzymes to improve sequence coverage for probing lysine microenvironments in IMP complexes.

Building upon our earlier efforts in characterizing IMP structure through mass spectrometry, we address here current gaps by developing a user-friendly method for IMP digestion, specifically tuned for IMP footprinting studies. This method involves filter-aided sample preparation (FASP), a method designed to effectively remove detergents and chaotropes that are not compatible with mass spectrometry or IMP digestion [44]. Extensive screening of conditions, including choice of surfactants, chaotropic reagents, and enzymes, was conducted to evaluate the IMP digestion performance. The choice of VKOR as a model was because it contains a hydrophilic outer membrane domain and a hydrophobic TM. These features enabled a direct comparison of peptide abundances between these domains, revealing their unique properties. These findings have promising implications for future analysis of IMPs, particularly when maximal sequence coverage for structural investigations is needed.

This protocol was initially developed to study transmembrane domains of the VKOR membrane protein [45], and it has been successfully adopted for other membrane protein structure studies with different footprinting techniques [45,46,47,48]. For non-footprinted VKOR protein, the optimized workflow using chymotryptic digestion typically yields sequence coverage above 90%. For footprinted IMPs, sequence coverage depends on the footprinting chemistry. Based on our previous studies, DEPC footprinting yields lower coverage (approximately 80%), whereas other reagents achieve coverage above 90% (see Table S2 in the Supporting Information). The details for developing this protocol, however, have not been reported yet. Our aim is to provide a comprehensive protocol, offering details in methodology development that have undergone systematic evaluation for IMP digestion. Satisfying our aim will include elucidating critical steps, troubleshooting problems, and acknowledging any limitations associated with the protocol. Nevertheless, this approach was successfully employed for different footprinting approaches, suggesting its potential for broad application in structural mass spectrometry.

## 2. Materials and Methods

### 2.1. Reagents and Materials

Unless otherwise noted, all materials were used as received from commercial sources without further purification. Tris base (>99.9% purity), urea, water, acetonitrile, and formic acid were obtained from Sigma-Aldrich Chemical Company (St. Louis, MO, USA). *n*-dodecyl-*β*-*D*-maltopyranoside (DDM) was obtained from Anatrace (Maumee, OH, USA). The membrane protein VKOR protein was provided by Dr. Weikai Li, Department of Biophysics at Washington University in St. Louis. Chymotrypsin and TCEP-HCl were purchased from Thermo Fisher Scientific (Waltham, MA, USA). Thermolysin, trypsin, pepsin, and ProteaseMAX™ surfactant were from Promega (Madison, WI, USA). RapiGest surfactant was purchased from Waters (Milford, MA, USA). The Microcon-30 kDa Centrifugal Filter Unit was purchased from Merck Millipore (formerly MilliporeSigma. St. Louis, MO, USA, catalog no. MRCF0R030). Centrifugation was performed using an Eppendorf 5425 R centrifuge (Enfield, CT, USA). The concentrations of all the protein stock solutions were determined by UV absorption using a Thermo Scientific™ NanoDrop™ (Wilmington, DE, USA).

### 2.2. VKOR Working Solution Preparation

The codon-optimized VKOR gene from *Synechococcus* sp. was expressed in *E. coli* with a C-terminal His tag. The recombinant protein was purified by Ni-affinity chromatography followed by gel-filtration chromatography, as described previously [49]. DDM (3 mg; Anatrace, D310) was dissolved in 10 g of Tris buffer (200 mM, pH 7.5) to prepare a 0.03% (*w*/*w*) solution, followed by brief sonication to ensure complete dissolution. The VKOR stock solution (1 mM) was then diluted with the 0.03% DDM buffer to obtain a 100 μM working solution. The 0.03% DDM buffer was prepared fresh daily. VKOR working solutions were aliquoted and stored at −80 °C for up to 6 months to avoid repeated freeze–thaw cycles.

### 2.3. Filter-Aided Sample Preparation for IMP Digestion

**Figure 1:**
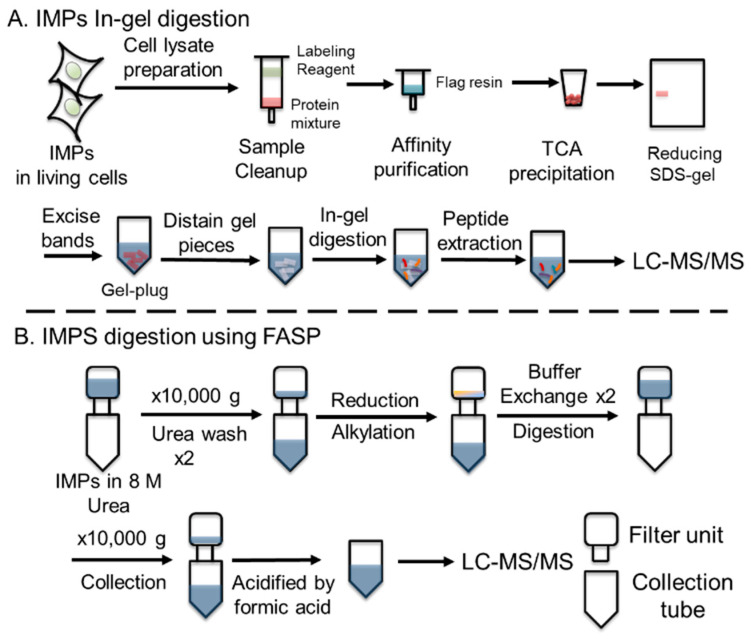
(**A**) Schematic of in-gel digestion for IMPs. After affinity purification, the IMPs are enriched using trichloroacetic acid (TCA) precipitation. Subsequent steps include electrophoresis and in-gel digestion to generate peptide fragments, followed by LCMS analysis. The entire workflow typically spans 3–4 days. (**B**) Schematic of IMP digestion using FASP. IMPs are washed with 8 M urea to dissociate lipids or detergents from vesicles. Alkylation of IMPs is performed in the presence of urea, followed by buffer exchange. The denatured IMPs are then digested to produce peptides suitable for LCMS analysis. The workflow can be completed within 1 day.

The FASP protocol was modified on the basis of the previous literature [50] (the workflow for FASP is described in Figure 1 in the main text). Specifically, ultrafiltration units containing 50 µL VKOR (10 µM) were added to 200 μL of denatured solution containing 8 M urea in 0.1 M ammonium bicarbonate (pH 8.0) in the ultrafiltration units and centrifuged at 10,000× *g* for ~20 min until less than 10 μL of sample remained above the filter. Based on a molecular weight of ~31 kDa for VKOR, this corresponds to approximately 0.5 nmol (∼15.5 µg) of VKOR protein loaded per filter. Then, 200 μL of denatured solution was added to the ultrafiltration units, and this wash step was repeated twice. The flow-through from the collection tube was discarded, and 100 μL 50 mM TCEP in 0.1 M ammonium bicarbonate was added to the filter and incubated at 37 °C for 30 min. Iodoacetamide (IAA, 100 μL, 100 mM) in 0.1 M ammonium bicarbonate was added, and the resulting solution was placed in the dark for 30 min. The ultrafiltration units were centrifuged at 10,000× *g* for ~20 min, and 150 μL of digestion buffer (DB, 0.1 M ammonium bicarbonate) was added to the filtration units, and they were centrifuged at 10,000× *g* for 10 min. This step was repeated twice. At this stage, urea and excess IAA and TCEP were removed by DB wash, and 60 μL of DB and protease (enzyme-to-protein ratio 1:20 (*w*/*w*)) was added. For pepsin digestion, 0.1 M HCl in water was used as the digestion buffer. The units were placed in a water bath at 37 °C overnight. Note: The incubation of ultrafiltration units in a thermomixer will result in evaporation of DB overnight. After digestion, the ultrafiltration units were transferred to new collection tubes, and they were centrifuged 10,000× *g* until the solution completely passed the filter membrane. A volume of 100 μL of DB was added, and the ultrafiltration units were centrifuged at 10,000× *g* until the solution completely passed the filter membrane. This step was repeated. The flow-through was collected, and 1 μL formic acid was added to acidify the solution. Proteolysis was halted by formic acid acidification and by removal of proteases via the ultrafiltration membrane. The flow-through containing proteolytic peptides was used for subsequent MS analysis. The concentration of the peptides was determined by using a Thermo Scientific™ NanoDrop™.

### 2.4. LC-MS/MS Mass Analysis

After digestion, a 20 μL aliquot of each sample was diluted to 40 μL with 0.1% formic acid (FA), and 5 μL was injected onto a custom-built fused-silica capillary column packed with C18 reversed-phase material (Waters Symmetry, 5 μm, 100 Å, 75 μm × 30 cm, Milford, MA, USA). Mobile phase A consisted of 0.1% FA in water, and mobile phase B consisted of 80% acetonitrile with 0.1% FA. Separate LC gradients were used for nonspecific proteases (chymotrypsin, pepsin, and thermolysin) and trypsin. For nonspecific digests, the gradient was 2.5% to 65% solvent B over 80 min, followed by an increase to 98% solvent B over 5 min at a flow rate of 0.5 μL/min, a 10 min wash at 98% solvent B, and re-equilibration at 2.5% solvent B for 15 min. For tryptic digests, which generate longer peptides containing hydrophobic transmembrane helices, an extended gradient of 2.5% to 80% solvent B over 140 min was applied, followed by the same wash and re-equilibration steps. Mass spectrometric analyses were performed on a Q Exactive Plus hybrid quadrupole-Orbitrap mass spectrometer equipped with a Nanospray Flex ion source (Thermo Fisher Scientific), operated in data-dependent acquisition mode with a spray voltage of 3.0 kV and a capillary temperature of 250 °C. Full MS scans were acquired in the Orbitrap over an *m*/*z* range of 300–2000 at a resolving power of 70,000 (at *m*/*z* 400), followed by MS/MS scans at a resolving power of 17,500; the 15 most abundant precursor ions were selected for HCD fragmentation with an AGC target of 200,000 and a dynamic exclusion duration of 5 s.

### 2.5. Data Analysis

LC-MS/MS raw files were imported into the Byonic™ Software (Protein Metrics, v5.9.5, San Carlos, CA, USA) for peptide mapping. Byonic™ processing of the LC-MS/MS data directly provided sequence coverage and the number of unique peptides under different proteolytic conditions. Chosen were a 10 ppm precursor mass tolerance, 20 ppm fragment mass tolerance, and CID/HCD fragmentation. The search parameters used were as follows: for trypsin, the digest mode is fully specific on K and R, and the “max missed” cleavages were five. Because chymotrypsin exhibits broadened cleavage behavior under denaturing structural proteomic conditions, as previously reported [45,51], a nonspecific database search strategy was applied for these nonspecific enzymes. When nonspecific proteases (i.e., chymotrypsin, thermolysin, pepsin) were used, the digest mode was set as nonspecific. To balance database completeness and search-space complexity, a two-step database search strategy was applied. First, a search without variable modifications was performed using a database containing VKOR, the *E. coli* proteome, contaminants, and decoys (protein FDR 1%). Proteins meeting a high-confidence threshold (log *p*-value at least 2.0 units lower than that of the top decoy protein [52]) were selected to construct a focused database. This reduced database retained the selected proteins together with contaminants and decoys and was then used for a second search including variable modifications. Carbamidomethylation (+57.0215) of Cys was set as fixed modification, oxidation (+15.9949 Da) of Met, Tyr, Phe, Trp, and carbamylation (+43.0058 Da) of the N-terminals of peptide, Thr, Ser and Lys were set as common modifications. The full Byonic search configuration is provided in the Supporting Information (Tables S5 and S6). The mass spectrometry proteomics data were deposited to the ProteomeXchange Consortium via the PRIDE [53] partner repository with the dataset identifier PXD072461.

## 3. Results and Discussion

### 3.1. FASP for VKOR Membrane Protein Digestion

Peptide IMPs, which are naturally embedded within lipid bilayers, rely on this environment for proper folding and functionality. Preserving their structure is vital for downstream analysis like mass spectrometry, X-ray crystallography, and functional assays. Researchers commonly employ detergents or lipid environments to mimic native conditions and to stabilize protein structures. This pursuit, however, adds complexities to sample preparation, mandating purification before digestion and subsequent MS analysis.

Previously, we developed an in-cell footprinting method to probe membrane protein−drug interactions in live cells [42]. The conventional urea–trypsin, in-solution digestion that is used for soluble proteins typically takes one day, but it proved inadequate for the digestion of IMPs. To address this challenge, we devised an in-gel digestion protocol, enabling the separation of individual bands or spots of IMPs. This separation is particularly advantageous when dealing with complex protein mixtures. Moreover, in-gel digestion not only enriches IMPs, but also reduces contamination, giving a cleaner peptide sample suitable for subsequent MS analysis. It is important to acknowledge that in-gel digestion does have limitations, including the potential loss of low-abundance hydrophobic peptides and the intricacy of gel handling. Additionally, the time required for the protocol, usually spanning several days, restricts experimental throughput (Figure 1A).

We hypothesized that FASP could serve as a viable approach to eliminate detergents or surfactants, thereby facilitating MS analysis of IMPs [44,54]. As depicted in Figure 1B, the FASP method comprises four crucial steps. First, undesirable low-molecular-weight contaminants (e.g., lipids, fatty acids, detergents) can be removed by washing with a urea-containing buffer. This clean-up step not only serves to eliminate detergents and lipids but also to unfold IMPs for denaturation and to increase their surface area for chemical modification and enzymatic digestion. Subsequently, denatured IMPs undergo reduction, and their free cysteine residues are capped through alkylation. Following buffer exchange to ensure compatibility with proteases, the IMPs are digested by a protease. Finally, the resulting peptides are collected for LC/MS analysis. Initially, we employed this methodology to digest the VKOR membrane protein, which yielded 80.6% sequence coverage by using tryptic digestion (Figure S1, Supporting Information). This approach shortens the time for sample preparation to one day and achieves VKOR sequence coverage comparable to that of in-gel digestion methods.

### 3.2. Evaluation of Protease and Additives for IMP Digestion

**Figure 2:**
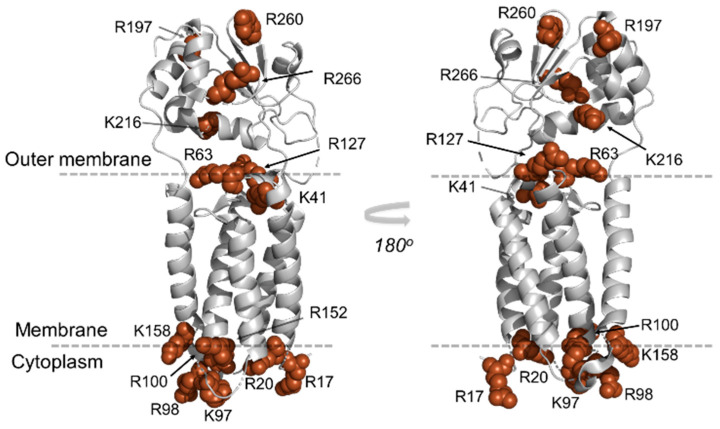
Mapping of tryptic cleavage sites (Arg and Lys) on the membrane protein VKOR crystal structure (PDB 3KP9). Tryptic residues in VKOR are found predominantly on the outer membrane Trx domain, the membrane interface, and the cytoplasmic side of the membrane, whereas the long α-helices in TMs are devoid of such tryptic cleavage sites.

Although trypsin remains a gold standard enzyme owing to its specific cleavage properties, minimal self-digestion, and generation of charged residues (Lys and Arg) at peptide C-termini—ideal for charge location, MS ionization, and fragmentation by MS/MS—it is important to note that conventional trypsin-centered strategies are less efficient for IMP digestion. This inefficiency arises because (1) TMs typically lack the charged Lys and Arg necessary for trypsin cleavage (Figure 2) and (2) tryptic transmembrane peptides tend to be both large and hydrophobic. These peptides adhere to plastic surfaces and often escape detection by mass spectrometers. Even when detected, they are frequently retained on reversed-phase columns, resulting in broad peaks that compromise quantification accuracy, as peak area is typically used for quantification. Further, their length hinders efficient fragmentation, yielding insufficient product ions to locate modification sites accurately and to achieve residue-level structural resolution. As a result, we achieved an average 80.6% sequence coverage with tryptic digestion (see peptide mapping in Figure S1, Supporting Information), the challenge remains to avoid longhydrophobic peptides from trypsin’s low cleavage sites in the membrane (see the representative tryptic transmembrane peptide (residues 101–127) in Figure S2, Supporting Information).

Next, we explored alternative nonspecific proteases, including chymotrypsin, thermolysin, and pepsin, to see if we can obtain smaller, more informative peptides (we screened proteases whose properties are summarized in Table S1, Supporting Informaiton). Chymotrypsin is a good choice because it preferentially cleaves at the C-terminal of aromatic amino acids Tyr, Phe and Trp at a high rate, and at Leu and Met at a lower rate. Thermolysin cleaves at the N-terminus of Leu, Phe, Val, Ile, Ala, and Met. Both enzymes maintain their enzymatic activity at pH 8.0, which is the standard pH for most proteomic studies. Pepsin has its highest activity between pH 2–4, and cleaves with limited specificity at the C-terminus of aromatic and hydrophobic residues Y, F, W and L, or even after A or G, its specificity being pH-dependent [55,56]. To date, pepsin is the most commonly used protease for HDX of membrane proteins [39] owing to the need to maintain enzymatic activity at pH = 2.5 [57], a pH where the protein has minimal back-exchange of D to H.

**Figure 3:**
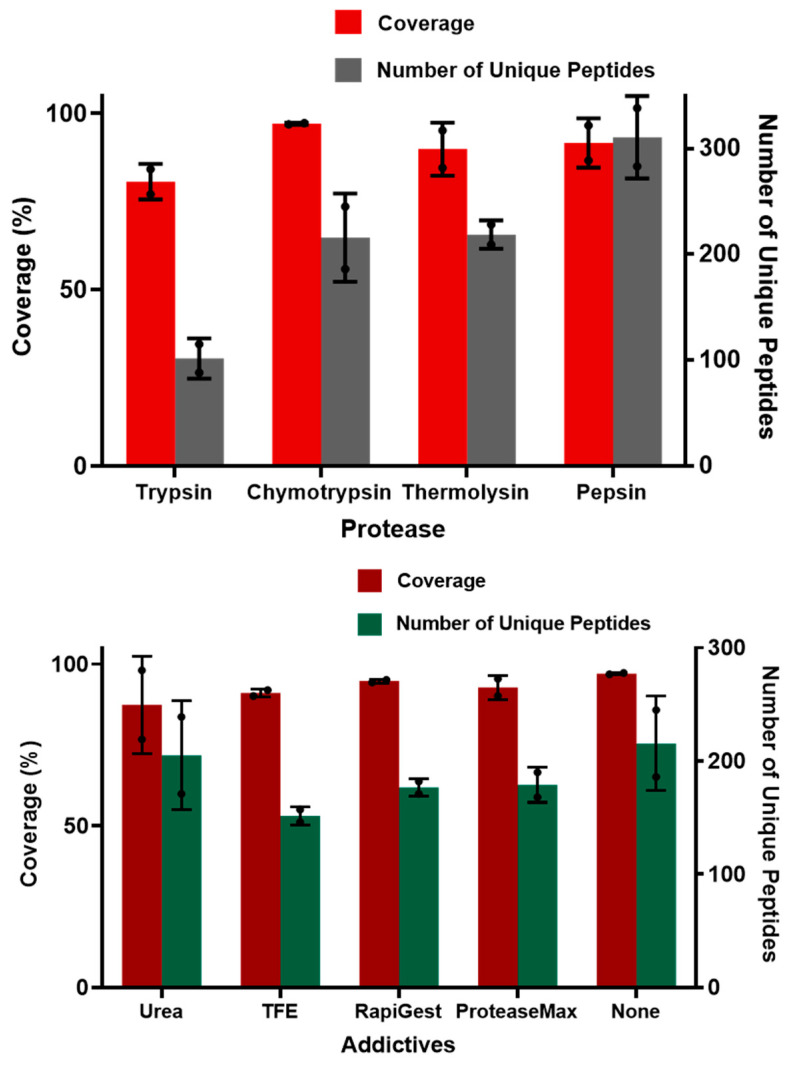
Screen of proteases (**top**) and additives (**bottom**) for digestion coverage of an IMP. Trypsin and chymotrypsin both achieved moderate to high sequence coverage, with average values of 80.6% and 97.0%, respectively. However, tryptic digestion generates long peptides within transmembrane regions owing to the scarcity of Lys and Arg residues, resulting in poor chromatographic resolution and broad LC peaks (See Figure S2). Chymotrypsin was thus selected as the optimized protease for subsequent experiments. The inclusion of additives did not improve digestion efficiency or sequence coverage. Chymotryptic digestion without additives yielded the highest average sequence coverage (97.0%), whereas the addition of urea, 10% TFE, 0.1% RapiGest, or ProteaseMAX resulted in reduced coverage (87.4%, 91.0%, 94.7%, and 92.8%, respectively).

We evaluated the number of unique peptides and sequence coverage for a set of enzymatic conditions (Figure 3). Chymotrypsin afforded the highest sequence coverage, with an average of 97.0%. On the contrary, pepsin generates a relatively high number of unique peptides but with average coverage (91.6%). Because pepsin is a nonspecific protease, it can cleave proteins into small pieces. Some peptides are so small that they are beyond the detection capacity for LC-MS systems owing to their high hydrophilicity or having an *m*/*z* too low to be included in the data-dependent acquisition (DDA) range setting.

Notably, chymotrypsin and thermolysin give comparable coverage but generate different peptides (Figure S3 and Table S1, Supporting Information). Chymotrypsin cleaves on the C-terminal side of amino acids whereas thermolysin cleaves at the N-terminal side. The complementary nature of these two enzymes is a promising feature for IMP bottom-up analysis if one wishes to generate overlapping peptides for high confidence and good spatial resolution. Indeed, we observe 99.6% sequence coverage with a combination of these two proteases, with only the methionine at the N-terminus not detected (Figure S3, Supporting Information).

Chemical additives provide effective means for IMP denaturation and solubilization. These agents have been widely employed in primary-structure proteomics to enhance IMP enrichment and digestion. For example, Veenstra and colleagues [58] utilized methanol, assisted by sonication, to identify over 700 IMPs, including peptides from transmembrane segments. In a separate study, Choolani and cowokers [19] found that trifluoroethanol (TFE) has a marked preference to release peptides with high hydrophobicity. Acid-labile detergents (e.g., rapigest [16,59]) were developed as a substitute for organic solvents or surfactants. These agents serve to solubilize proteins and simplify detergent removal before LC-MS analysis.

Interestingly, our results indicate that the inclusion of organic solvents or acid-labile detergents does not improve sequence coverage for single IMP footprinting when using a FASP-based workflow. Under these conditions, sequence coverage was comparable, and in some cases reduced, relative to additive-free digestion (Figure 3 (bottom)). This outcome can be attributed to several features of the experimental design. First, extensive protein denaturation and solubilization are already achieved during FASP processing, as VKOR is treated with 8 M urea to disrupt membrane association, followed by reduction of disulfide bonds with TCEP and subsequent alkylation, resulting in effective and largely irreversible denaturation prior to enzymatic digestion. Second, protein footprinting experiments typically focus on a single purified protein or protein complex, substantially reducing sample complexity and dynamic range compared to proteome-scale analyses. Third, protein input can be easily controlled in the FASP workflow; in this study, more than 15 µg of purified protein was routinely applied, ensuring sufficient peptide yield for LC–MS analysis even in the presence of partial sample loss. Collectively, these considerations explain why additional solubilizing agents provide limited benefits in this context and may instead introduce unnecessary chemical complexity or adversely affect digestion efficiency and downstream footprinting measurements. These considerations underscore the importance of matching digestion and solubilization strategies to the experimental context.

We found that the use of urea is less potent for IMP digestion. Even with 2 M urea, we observed decreased coverage (87.4%) compared to other conditions (Figure 3). It should be noted that commonly used chaotropic reagents, such as 1 M guanidine and 2 M urea, are compatible with most proteases. Our results suggest that 2 M urea may reduce enzymatic ability and hinder achieving full coverage. Our result is consistent with that of the Heck group [12] who found no benefits of 2 M urea for trypsin digestion efficiency in terms of the identified IMPs. In short, chymotrypsin digestion without any additives is the most favored proteolytic condition.

Finally, the presence of background proteins from the *E. coli* expression system was further investigated. A preliminary database search identified 107 proteins in the chymotrypsin-digested samples, including host cell proteins and common contaminants frequently co-purified with membrane proteins. In the protein score ranking (Figure S7 and Table S7), chymotrypsin was ranked highest, followed by VKOR, despite being introduced at a lower proportion relative to VKOR (1:20, *w*/*w*, chymotrypsin:VKOR). This observation may reflect differences in peptide detectability rather than relative protein abundance. As a soluble protein, chymotrypsin can undergo autoproteolysis and generate readily detectable peptides, whereas VKOR, as an IMP, produces more hydrophobic peptides that are generally less amenable to LC–MS/MS analysis because of reduced ionization efficiency and detectability.

### 3.3. The “Split Personality” of IMPs for Routine LCMS Analysis

IMPs are amphipathic—often composed of hydrophilic extramembrane segments and hydrophobic transmembrane segments that occupy the phospholipid bilayer. Recognizing this “split personality”, we next test how these two-segment peptides respond in MS detection.

**Figure 4:**
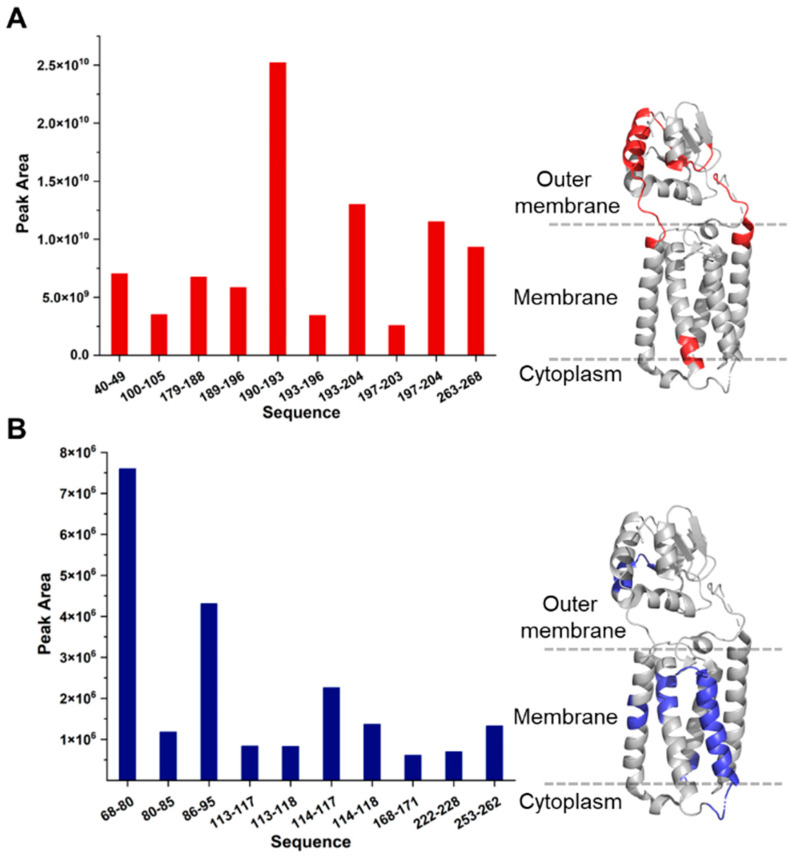
The abundances of selected peptides: (**A**) The EIC peak area for the 10 most abundant chymotryptic peptides. (**B**) The EIC for the 10 least abundant chymotryptic peptides. Nine of the ten lowest abundance peptides come from hydrophobic TMs, whereas the 10 most abundant peptides all come from the outer membrane or membrane interface. TMs are located between the outer membrane and cytoplasmic regions and are indicated by dashed lines in the structure. The selected peptides are mapped onto the VKOR crystal structure (PDB 3KP9).

The bacterial form of VKOR, which is naturally fused to a Trx-like domain, contains 285 amino acids. Its crystal structure [49] shows five membrane-embedded α-helices followed by a linker segment to the extracellular Trx-like domain. Theoretically, the peptides from digestion should occur with equal relative molar ratios if there is no miscleavage. This is because each VKOR protein can generate only one copy of a peptide from a specific region, regardless of whether the peptides are derived from the outer membrane region or the TMs. We examined the extracted ion chromatograms (EICs) for 20 signature peptides, including the 10 most abundant and the 10 least abundant (Figure 4). The comparison reveals an unrecognized trend in membrane proteomics. The top 10 most abundant peptides all originate from the extramembrane region or the interface between the outer membrane and transmembrane. Conversely, among the 10 least abundant peptides, nine come from the transmembrane region. The EIC comparison between the most and least abundant peptides spans over three orders of magnitude. This finding provides direct evidence for the “split personality” of IMPs.

As expected, the eight most abundant peptides contain positively charged residues that enhance solubility and ionization efficiency. In contrast, the 10 least abundant peptides lack these positively charged residues. The scarcity of positively charged residues in transmembrane regions contributes to their underrepresentation even within the same individual IMP. Current LC-MS systems are programmed to favor peptides containing positively charged amino acids in the data-dependent mode. Chymotryptic peptides from the TMs are intrinsically hydrophobic because they are devoid of charged residues, resulting in poor ionization efficiency and increased surface adsorption, and, thus, they are not abundant.

### 3.4. Identification of “Sweet Spot” for Transmembrane Peptide Detection

**Figure 5:**
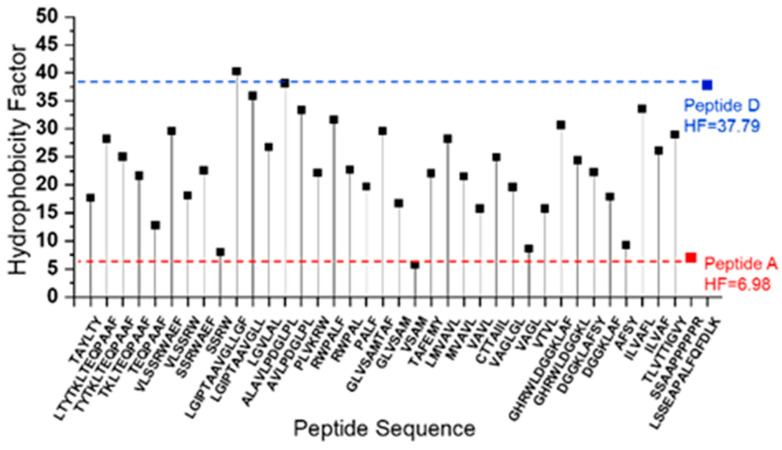
Identification of the “sweet spot” for transmembrane peptide detection. Peptide A and D are the most hydrophilic and most hydrophobic peptides, respectively, from Pierce peptide retention time calibration mixture. The calculation defines a “sweet spot” between the blue line (maximum hydrophobic line defined by peptide D) and red line (maximum hydrophilic line defined by peptide A). We consider the HF for most detected peptides from transmembrane as the “sweet spot”.

Our next goal was to determine the compatibility of peptides for MS detection. Reversed-phase chromatographic approaches are suited for peptides falling within a certain window of hydrophobicity. We hypothesize that peptide standards with a broad range of hydrophobicity would allow us to evaluate the instrument’s capability to detect peptides spanning various hydrophobicity levels in IMPs. We utilize Pierce’s peptide retention time calibration (PRTC) mixture that comprises 15 heavy peptides with a broad hydrophobicity. Hydrophobicity values were calculated using the Thermo Fisher peptide analyzing tool [60,61]. We refer to this predicted hydrophobicity value as the hydrophobicity factor (HF). Using the online tool, we calculated the hydrophobicity for both the most hydrophobic and most hydrophilic peptides among the 15 heavy peptides, as well as for all the detected chymotryptic peptides from VKOR’s TM (Table S4, Supporting Information). A workflow for the peptide hydrophobicity calculation is provided in the Supporting Information (Figure S5). As an example, for the first peptide shown in Figure 5 (sequence: TAYLTY), the sequence was entered into the input field and analyzed by using the tool, yielding a hydrophobicity factor of 17.73, as shown in Figure S6 in the Supporting Information. Using the workflow described above, we calculated HF values for all validated peptides, and they are summarized in the Supporting Information Table S4. The calculated values define a “sweet spot” between the most hydrophobic peptide and most hydrophilic peptide (see region between blue line and red line in Figure 5). Notably, most detected peptides from the transmembrane region fall within this “sweet spot.” Further, over half of the identified transmembrane peptides exhibit an HF exceeding 20. This “sweet spot” offers guidance to choose an appropriate protease that cleaves IMPs into peptides falling in the “sweet spot” region. As an illustrative strategy, the investigator can conduct an in silico digestion and then predict the HF of the peptide generated. The prevalence of peptides within the “sweet spot” is a measure of a favorable potential for peptide detection.

**Figure 6:**
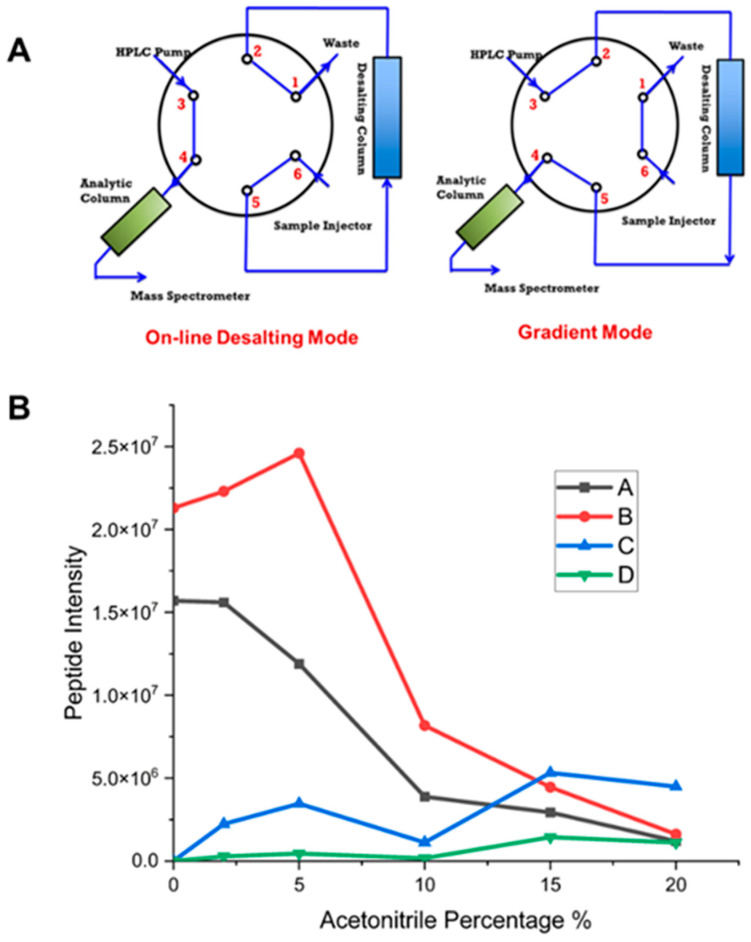
(**A**) The sample-loading valve system operates in two modes. In the desalting mode, the sample is directed to a desalting column connected to waste, effectively removing salts, contaminants, and hydrophilic peptides. After desalting, the system shifts to gradient mode. In this mode, the HPLC pump elutes the analyte from the desalting column to the analytical column for mass analysis. (**B**) The peptide signal intensity is shown corresponding to acetonitrile percentage. A (HF = 7.0) and B (HF = 11.8) represent the two most hydrophilic peptides in PRTC, while C (HF = 36.1) and D (HF = 37.8) represent the two most hydrophobic peptides in PRTC.

The detection of transmembrane peptides within the “sweet spot” is partly attributed to our two-mode, sample-loading valve system (Figure 6A). The proteolytic peptides in the buffer solution possess several salts, detergents, and other adduct-forming ions. These can interfere with MS characterization by decreasing sensitivity and can result in instrument fouling, ultimately leading to downtime. To improve the MS sensitivity, on-line desalting valves were applied in our LC-MS system to remove salts and other buffer components prior to MS detection. Ideally, the peptide sample was loaded into the desalting column to remove hydrophilic matrix components (send to waste) while trapping peptides of interest. To ensure sufficient sample loading and desalting, we employed a 10 min elution with aqueous phase.

We assessed the capabilities of the LC-MS/MS system by using PRTC as our benchmark. PRTC was prepared in varying acetonitrile concentrations. Representative peptides A, B, C, and D were monitored for their mass response relative to acetonitrile concentration. We observed a binary mass response corresponding to organic solvent (Figure 6B). First, the signal intensities corresponding to the most hydrophobic peptides C and D generally increased when we increased the concentration of acetonitrile (see blue and green curve). This observation is consistent with the principle that organic solvents facilitate hydrophobic peptide solubilization and with the results by Mitra et al. [62] who observed a higher percentage of membrane vs. non-membrane proteins when utilizing methanol solubilization. The curve does not show, however, a strictly positive correlation with the acetonitrile percentage. Notably, the intensity of peptide C decreases at 10% acetonitrile but increases again at 15% acetonitrile. On the other hand, an opposite trend occurred for the most hydrophilic peptides A and B whose signal intensities declined with an increasing concentration of acetonitrile. These two hydrophilic peptides decreased in signal intensity with increasing acetonitrile likely owing to poor retention on the desalting column and leading to partial elution to waste. This loss may shed light on the diminishing sequence coverage seen with an increasing number of unique peptides in pepsin digestion (Figure 3 (top)). Pepsin’s broad specificity may lead to digestion of VKOR into smaller peptides or even amino acids that cannot be captured by the desalting column, ultimately leading to a decrease in sequence coverage. These results also underscore the significance of finely tuning the LC-MS system. For instance, by introducing organic solvents to dissolve the digested peptide, one can tailor the “sweet spot” to prefer either hydrophilic (extramembrane) or hydrophobic (transmembrane) segments.

### 3.5. Application of Method to IMP Structure Analysis by Footprinting

**Figure 7:**
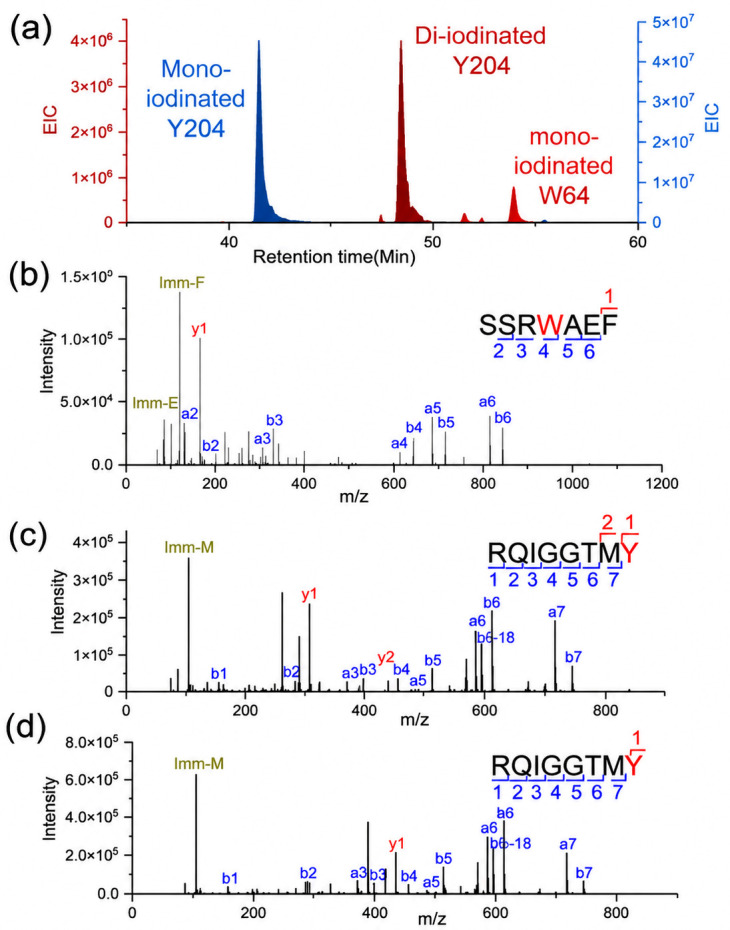
Representative product-ion (MS/MS) spectra for chymotryptic peptides. (**a**) Extracted ion chromatograms for modified peptides. (**b**) Product-ion (MS/MS) spectra of mono-iodinated W64, (**c**) mono-iodinated Y204, and (**d**) di-iodinated Y204. Chymotrypsin displays a preference for cleaving at the C-terminal side of aromatic amino acid residues (e.g., W and Y, generating a peptide amenable for peptide sequencing). Panels (**c**) and (**d**) include MS/MS spectra of the same peptide previously reported in our published work [45]. They are shown here to illustrate how these iodinated peptides, including the iodinated Tyr and Trp-containing peptide, are resolved by LC.

Protein footprinting can provide residue-level structural information. Achieving this resolution, however, relies on the ability to identify and quantify unambiguously the peptides derived from protein digestion and to annotate modifications that are induced by footprinting. For structural analysis, two critical questions come into play: (i) are there adequate fragment ions to pinpoint the modification site, and (ii) are the chromatographic peak shapes sufficiently well-formed and separated to quantify the modification ratio by integrating extracted ion chromatograms (EICs) of precursor ions. Given the nature of IMPs, answering these questions is a significant challenge through conventional sample handling. In Figure 7, a chymotryptic peptide is sufficiently fragmented to confidently identify the iodination on Y204 and W64. With tryptic digestion, however, a long transmembrane peptide is liberated, leading to poor resolution in peptide sequencing and chromatographic lipid chromatogram separation. This degradation in resolution adversely affects obtaining structural information and undermines the accuracy of quantification (Figure S2). We tested the current approach to analyze the protein after footprinting by different reagents. Coupled with novel footprinting, the approach was successfully applied to study two IMPs including human VKOR and GLUT1 proteins. Importantly, this approach is applicable to several footprinting methods, including iodination [45], DEPC [48], and photo oxidation [47] and consistently yields sequencing coverage exceeding 90%.

## 4. Conclusions

We developed an approach to IMP digestion suitable for a nearly complete structure analysis (e.g., as for protein footprinting). The method employs commercially available, microcentrifuge filtration devices (spin filters) to be used in washing away contaminating species prior to MS analysis. The method greatly reduces the time required for sample preparation, minimizes instrument contamination, and provides nearly full MS sequence coverage of IMPs. We evaluated four different proteases (i.e., trypsin, chymotrypsin, thermolysin, and pepsin) and five additives (i.e., organic solvents, chaotropes, and acid-labile surfactants), and found that additives are not necessary if FASP is applied. Aided by a retention time calibration mixture and HF simulation, we utilized a “sweet spot” that determines the capacity for our LC-MS system to deal with the wide range of hydrophobic and hydrophilic peptides from IMPs. The outcome provides guidance for choosing a suitable protease in IMP structural proteomics.

There are still challenges and limitations of our developed protocol. First, chymotrypsin is a nonspecific protease. This introduces complexity to the peptide mixture and can complicate accurate determination of modification ratios. This downside, however, can be mitigated in two ways. (i) Despite its propensity to cleave at various sites, chymotrypsin displays a preference for specific residues (such as Trp, Tyr, Phe, and to a lesser extent Leu, Met, and His). This allows us to choose the most abundant peptide as representative to quantify the modification ratio. Furthermore, in structural analysis, the need for absolute peptide quantification is often unnecessary when the goal is to compare the same peptide from a single protein in two different states. The systematic error resulting from unspecific digestion may cancel, provided that the modification does not alter the digestion pattern. (ii) Most footprinting experiments are performed for a single protein complex. The identification of non-specifically cleaved peptides is readily manageable with modern software and instrumentation. Additionally, the fast speed of modern MS and high-resolution chromatography enable sufficient scanning space for confident peptide identification.

Another limitation pertains to the FASP approach. Although FASP has been described as a universal method enhancing membrane protein identification in proteomics, issues concerning peptide/protein loss were reported [44,54,63]. For example, the original FASP study using purified bovine serum albumin (BSA) reported high peptide recovery while noting losses due to nonspecific adsorption to the filter membrane and device surfaces. Subsequent benchmarking studies employing purified protein standards, including GFP, reported overall peptide recoveries of approximately 90–95% under optimized conditions, whereas other comparative analyses and device evaluations observed lower recoveries (down to ~60–70%), particularly when surface adsorption is not mitigated. Consistent with these observations, enhanced FASP variants incorporating surface passivation strategies demonstrated that a substantial fraction of peptide loss arises from nonspecific binding within the ultrafiltration unit. In addition, low molecular weight cutoff (MWCO) filters may be susceptible to clogging caused by protein aggregation or residual debris, which can further compromise throughput and recovery. Quantitative assessment of peptide loss and potential filter-associated bias in FASP can be achieved using spike-in standards spanning a broad range of hydrophobicity. In such designs, synthetic or isotope-labeled peptides are added prior to FASP processing and compared with non-FASP controls to determine absolute recovery. Although absolute peptide recovery was not directly quantified in the present study, the use of a purified membrane protein system, strongly denaturing conditions, and multiple wash and collection steps likely reduced filter clogging and minimized sample-dependent variability. Future work will incorporate defined hydrophobic and hydrophilic peptide spike-ins to benchmark peptide recovery systematically and to further optimize the FASP-based workflow.

Although this protocol was evaluated using a purified single IMP, its underlying principles, strong denaturation, efficient detergent removal by FASP, and flexible protease selection, are not protein-specific and may be applicable to a broad range of IMPs. To date, we have successfully applied this approach to two IMP systems by using three different footprinting methods (see Table S2 in the Supporting Information), supporting its general utility for targeted structural proteomics [45,47,48,64]. With appropriate optimization, the workflow could also be extended to more complex IMP mixtures, such as enriched membrane fractions or affinity-purified protein complexes, where effective detergent removal is particularly critical for maintaining MS performance. In such cases, increased sample complexity will necessitate additional consideration of MS acquisition strategies, chromatographic separation, and data analysis workflows. In this scenario, alternative acquisition modes, including data-independent acquisition (DIA) and targeted parallel reaction monitoring (PRM), could be considered, as they can offer improved consistency and sensitivity for predefined peptide sets, including hydrophobic peptides. Nevertheless, the strategy described here may serve as a practical starting point for adapting digestion workflows in broader structural proteomic studies of IMPs. We further anticipate that this protocol will be compatible with other structural proteomic applications, including those that employ additional footprinting chemistries and cross-linking approaches.

## Data Availability

The mass spectrometry proteomics data were deposited to the ProteomeXchange Consortium via the PRIDE [53] partner repository with the dataset identifier PXD072461.

## Supplementary Materials

The following supporting information can be downloaded at: https://www.mdpi.com/article/10.3390/membranes16060215/s1. It includes Figure S1: Peptide sequencing coverage for tryptic digestion of VKOR; Figure S2: Tryptic peptide (101–127) from VKOR; Figure S3: Sequence coverage for chymotrypsin and thermolysin digestion; Figure S4: Extracted-ion chromatogram (EIC) of retention peptide standards; Figure S5: Workflow for calculation of peptide hydrophobicity factor (HF); Figure S6: Example of calculating HF of peptide TAYLTY; Figure S7: Protein score plot of identified proteins ranked by log p-value; Table S1: Screened proteases for IMP digestion; Table S2: Comparison of sequence coverage across different protein footprinting methods; Table S3: PFIPI footprinting modification percentages and standard deviations; Table S4: Calculation of peptide hydrophobicity factor; Table S5: Byonic search configuration for nonspecific enzyme digestion; Table S6: Byonic search configuration for trypsin digestion; Table S7: Proteins identified from the initial database search and used for construction of the focused database.
