# Supplementary material for: Optimized Digestion Conditions for Membrane Protein Footprinting and Mass Spectrometry Analysis
Source: Membranes (Basel). 2026 Jun 22;16(6):215. doi: 10.3390/membranes16060215 (PMC13304333; doi:10.3390/membranes16060215)
Supplement: Supplementary file 1 [file membranes-16-00215-s001.zip › membranes-4035526-supplementary.pdf]

Optimized Digestion Conditions for Membrane Protein Footprinting and Mass Spectrometry Analysis

Ming Cheng [1,2,*], Xinzhu Li [3], Lin Bai [1], Weikai Li [4] and Michael L. Gross [3,*]

[1] Shandong Laboratory of Yantai Drug Discovery, Bohai Rim Advanced Research Institute for Drug Discovery, Yantai 264117, China
[2] Shanghai Institute of Materia Medica, Chinese Academy of Sciences, Shanghai 201203, China
[3] Department of Chemistry, Washington University, St. Louis, MO 63130, USA; li.xinzhu@email.wustl.edu
[4] Department of Biochemistry and Molecular Biophysics, Washington University, St. Louis, MO 63130, USA
* Correspondence: chengming@simm.ac.cn (M.C.); mgross@wustl.edu (M.L.G.)

Supporting Information

Table of Contents

1. General information 2

2. Table S1. The screened protease for IMPs digestion.. 2

3. Experimental Results 2

3.1. Table S2. Comparison of sequence coverage across different protein footprinting methods 3

3.2. Table S3. PFIPI footprinting modification percentages and standard deviations 4

3.3. The sequence coverage for tryptic digestion.. 4

3.4. Selected tryptic transmembrane peptide 4

3.5. The sequence coverage for chymotrypsin and thermolysin digestion 4

3.6. The extracted-ion chromatogram (EIC) of retention peptide standard 5

3.7. Table S4. The calculation of hydrophobicity factor.. 5

3.8. Table S5. Byonic search configuration for nonspecific enzyme digestion 7

3.9. Table S6. Byonic search configuration for trypsin digestion.. 9

3.10. Figure S5. Workflow for calculation of peptide hydrophobicity factor (HF). 11

3.11. The example of calculating HF of peptide TAYLTY…………………………………….....12

3.12. Background protein analysis……………………………………………………………14

1. General information

Unless otherwise noted, all materials were used as received from commercial sources without further purification. Tris base (>99.9% purity), urea, water, acetonitrile, formic acid were obtained from Sigma-Aldrich Chemical Company (St. Louis, MO, USA). n-Dodecyl-β-D-Maltopyranoside (DDM) was obtained from Anatrace. The membrane protein VKOR protein was provided by Dr. Weikai Li, Department of Biophysics at Washington University in St. Louis. Chymotrypsin and TCEP-HCl were purchased from Thermo Fisher Scientific. Thermolysin, trypsin, pepsin, ProteaseMAX™ surfactant are obtained from Promega. RapiGest surfactant was purchased from Waters. Microcon-30kDa Centrifugal Filter Unit was from Millipore Sigma. The concentrations of all the protein stock solutions were determined by UV absorption by using a Thermo Scientific™ NanoDrop™.

2. Table S1. The screened protease for IMPs digestion

Protease	Family	Cleavage Site	Optimal pH value	Advantages	Limitations
**Trypsin**	Serine protease	C-terminal of Arg and Lys	8.0	Efficient and specific; Easy ionization and CID fragmentation.	The absence of positively charged residues (K, R) at TMs; the presence of negatively charged amino acids such as D, E prevents tryptic cleavage.
**Chymotrypsin**	Serine protease	C-terminal of Phe, Tyr, Leu, Trp and Met	8.0	Orthogonal to that of trypsin; preference for hydrophobic amino acids	Lower specificity; the efficiency of chymotrypsin toward different hydrophobic amino acid residues varies
**Pepsin**	Aspartic protease	C-terminal of Tyr, Phe and Trp	1.0-3.0	Remains active at low temperature (4 °C) and pH (2.5), which is essential for HDX experiments.	Lower and pH-dependent specificity. Pepsin has a preference for aromatic residues (Y, F and W) and L
**Thermolysin**	Metalloprotease	N-terminus of Leu, Phe, Val, Leu, Ala, Met	8.0	Thermostable metalloproteinase (70-95 °C). The high digestion temperatures may be used as an alternative to denaturants.	Lower specificity; Mass parameters are needed to be tuned to analyze short peptides.

3. Experimental Results

3.1. Table S2. Comparison of sequence coverage across different protein footprinting methods

Footprinting reagents	Protein system	Sequence coverage	Reference
Perfluoroisopropyl Iodide	VKOR	~97%	Angew. Chem. Int. Ed. 2021, 60, 8867
4-iodobenzyl alcohol	VKOR	~99%	J. Am. Soc. Mass Spectrom. 2023, 34, 12, 2700
DEPC	VKOR	~80%	J. Am. Soc. Mass Spectrom. 2021, 32, 11, 2636
NanoPOMP (OH radical footprinting)	VKOR	~100%	Nat Commun. 2021, 12, 7270
NanoPOMP (OH radical footprinting)	Glut1	~100%	Nat Commun. 2021, 12, 7270

3.2. Table S3. PFIPI footprinting modification percentages and standard deviations

Residue Number	Mono-iodination (%)		Di-iodination (%)		Standard D	
					Mono-iodination	Di-iodination
Y4	13.6	13.5	80.2	80.4	0.07	0.14
Y36	17.8	17.6	12.5	13.2	0.14	0.49
Y39	47.5	46.9	3.8	3.76	0.42	0.03
Y117	10.1	10.2	0.62	0.63	0.07	0.01
Y120	78.7	78.7	21.3	21.3	0.00	0.00
Y132	86.6	86.9	13.4	13.1	0.21	0.21
Y163	0	0	0.6	0.54	0.00	0.04
Y178	14.4	14.8	2.28	2.28	0.28	0.00
Y204	2.1	2.1	0.16	0.16	0.00	0.00
Y207	19.3	19.4	74.3	73.6	0.07	0.49
Y228	82.3	83.8	7.7	7.93	1.06	0.16
Y252	2.06	2.08	0.05	0.06	0.01	0.01
Y262	75.3	73.4	1.22	1.18	1.34	0.03
Y277	9.12	9.43	3.79	4.53	0.22	0.52

3.3. The sequence coverage for tryptic digestion

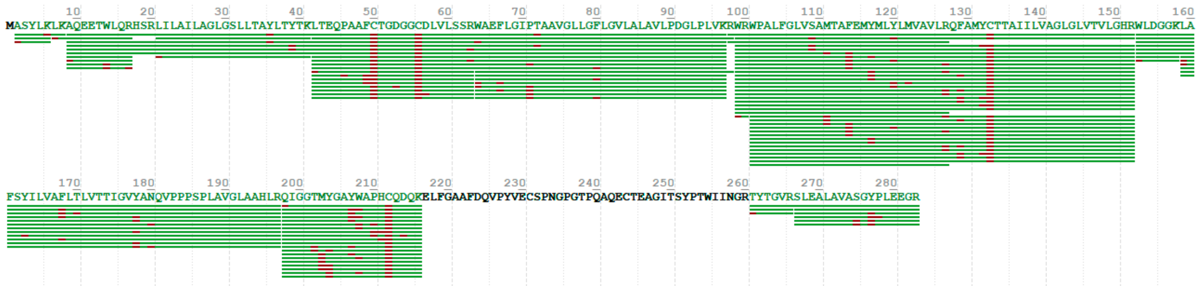

Figure S1. Peptide sequencing coverage for tryptic digestion of VKOR. Peptide search yielded a sequence coverage of around 80.6%. Tryptic peptides containing transmembrane segments generate large, hydrophobic peptides. One example is shown in Figure S2.

3.4. Selected tryptic transmembrane peptide

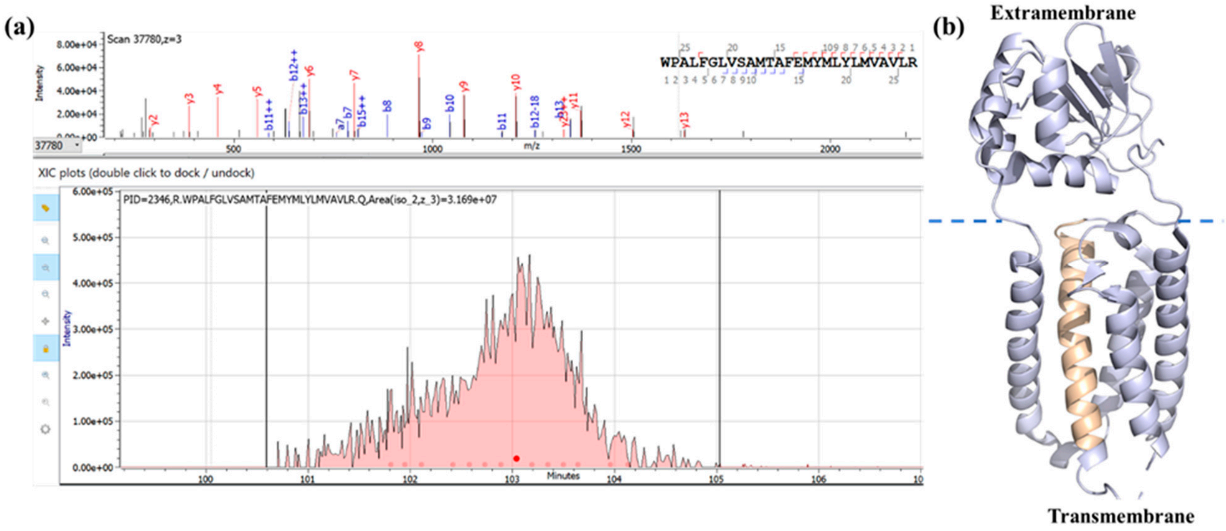

Figure S2. Tryptic peptide (101-127) from VKOR. (a) The MS2 spectra (top) and LC peak (bottom) for peptide 101-127. The peptide is eluted showing a broad peak ranging from 101-105 min. (b) Peptide (101-127) mapped on the X-ray crystal structural locates the peptide at the transmembrane domain.

3.5. The sequence coverage for chymotrypsin and thermolysin digestion

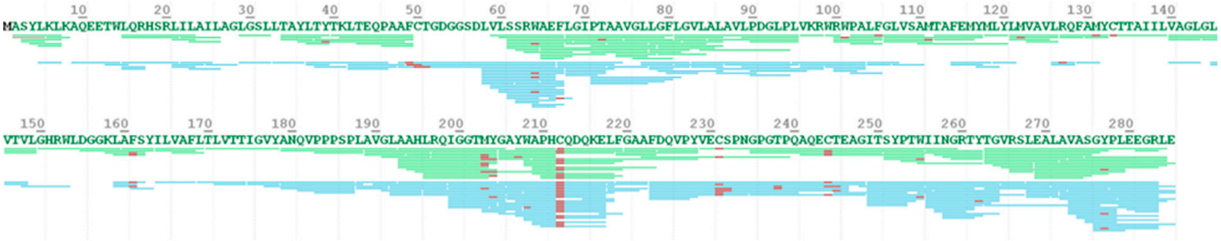

Figure S3. The sequence coverage for chymotrypsin (in green) and thermolysin (in blue) digestion. The two proteases generate two sets of overlapped peptides that increase the confidence for peptide identification. The combined outcome of two proteases is nearly full sequence coverage (99.6%). Red dots represent modifications on residues (e.g., oxidation, carbamidomethylation or carbamylation).

3.6. The extracted-ion chromatogram (EIC) of retention peptide standard

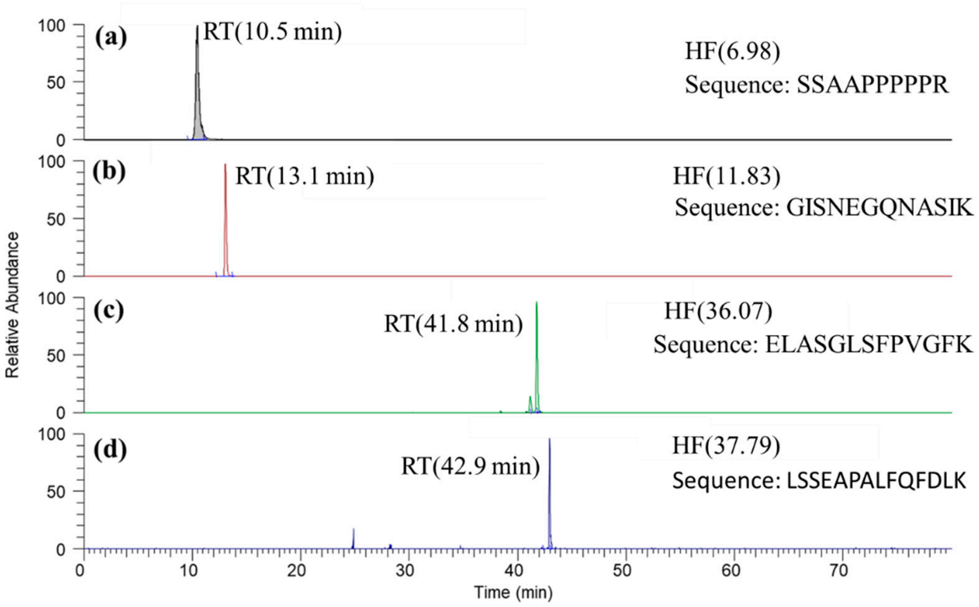

Figure S4. The extracted-ion chromatogram (EIC) of the two most hydrophilic peptides A and B, and the two most hydrophobic peptides C and D. The hydrophobicity factor was calculated (see table S4) to define the sweet spot for transmembrane peptide detection.

3.7. Table S4. The calculation of hydrophobicity factor

Peptide resource	Peptide sequence	Hydrophobicity factor
Chymotryptic VKOR digest	TAYLTY	17.73
	LTYTKLTEQPAAF	28.24
	TYTKLTEQPAAF	25.04
	TKLTEQPAAF	21.66
	TEQPAAF	12.79
	VLSSRWAEF	29.62
	VLSSRW	18.12
	SSRWAEF	22.54
	SSRW	8

	LGIPTAAVGLLGF	40.29
	LGIPTAAVGLL	35.93
	LGVLAL	26.77
	ALAVLPDGLPL	38.18
	AVLPDGLPL	33.32
	PLVKRW	22.19
	RWPALF	31.64
	RWPAL	22.66
	PALF	19.68
	GLVSAMTAF	29.59
	GLVSAM	16.68
	VSAM	5.72
	TAFEMY	22.02
	LMVAVL	28.21
	MVAVL	21.57
	VAVL	15.75
	CTTAIIL	24.94
	VAGLGL	19.57
	VAGL	8.58
	VTVL	15.75
	GHRWLDGGKLAF	30.67
	GHRWLDGGKL	24.41
	DGGKLAFSY	22.22
	DGGKLAF	17.84
	AFSY	9.23
	ILVAFL	33.53
	ILVAF	26.14

	TLVTTIGVY	28.98
Pierce™ Peptide Retention Time Calibration Mixture	SSAAPPPPPR (peptide A)	6.98
	LSSEAPALFQFDLK (peptide D)	37.79

The hydrophobicity factor is calculated by an online tool from thermos fisher scientific (Peptide Synthesis and Proteotypic Peptide Analyzing Tool).

3.8. Table S5. Byonic search configuration for nonspecific enzyme digestion

Recorded protein search configuration:		
Num	Rule	Value
0	Protein database	E:\Xinzhu\20260406_Ming Search2\Processing\23_Byonic_Selected Database\Nonspecific_Chymo.presets\proteins.fasta
1	Spectrum-level FDR	Auto cut
2	Cleavage residues	
3	Digest cutter	C-terminal cutter
4	Peptide termini	Non specific
5	Maximum number of missed cleavages	3
6	Fragmentation type	NA
7	Precursor tolerance	10.0 ppm
8	Fragment tolerance(CID)	20.0 ppm
9	Fragment tolerance(HCD)	20.0 ppm
10	Charges applied to charge-unassigned spectra:	1,2,3
11	Precursor mass max	10000.0
12	N-glycan search	None
13	O-glycan search	None
14	Off by x isotopes	-2,-1,0,+1,+2

15	Contaminants added	true
16	Decoys added	true
17	%Additional parameters:	
18	Disulfide Enable	false
19	Trisulfide Enable	false
20	DSS Crosslink Enable	false
21	Custom Crosslink Enable	false
22	Wildcard Enable	false
23	Combyne cut off score	Auto
24	Protein FDR cutoff	1%
25	Focused DB created	false
26	Export mzIdentML	false
27	Score version	2
28	precursor_assignment_flags	2
29	po_NumberMonosReturn	2
30	Lock mass list	None
31	do_quick_rejects	0
32	%Modification searches:	
33	common_modifications_max	2
34	rare_modifications_max	0
35	%Fixed and variable modifications:	
36	Oxidation / +15.994915 @ F, M, W, Y | common1	
37	Carbamyl / +43.005814 @ NTerm, K, S, T, Y | common1	
38	Carbamidomethyl / +57.021464 @ C | fixed	
39	% Custom modification text below	
40	%Glycan modifications:	

41	Show all N-glycopeptides	0
42	%Addition parameters:	
43	Product Version	PMI-Byonic-Demo:v5.9.5-ga0a5094d

3.9. Table S6. Byonic search configuration for trypsin digestion

Recorded protein search configuration:		
Num	Rule	Value
0	Protein database	E:\Xinzhu\20260406_Ming Search2\Processing\23_Byonic_Selected Database\Trypsin_rare0.presets\proteins.fasta
1	Spectrum-level FDR	Auto cut
2	Cleavage residues	RK
3	Digest cutter	C-terminal cutter
4	Peptide termini	Fully specific
5	Maximum number of missed cleavages	5
6	Fragmentation type	NA
7	Precursor tolerance	10.0 ppm
8	Fragment tolerance(CID)	20.0 ppm
9	Fragment tolerance(HCD)	20.0 ppm
10	Charges applied to charge-unassigned spectra:	1,2,3
11	Precursor mass max	10000.0
12	N-glycan search	None
13	O-glycan search	None
14	Off by x isotopes	-2,-1,0,+1,+2

15	Contaminants added	true
16	Decoys added	true
17	%Additional parameters:	
18	Disulfide Enable	false
19	Trisulfide Enable	false
20	DSS Crosslink Enable	false
21	Custom Crosslink Enable	false
22	Wildcard Enable	false
23	Combyne cut off score	Auto
24	Protein FDR cutoff	1%
25	Focused DB created	false
26	Export mzIdentML	false
27	Score version	2
28	precursor_assignment_flags	2
29	po_NumberMonosReturn	2
30	Lock mass list	None
31	do_quick_rejects	0
32	%Modification searches:	
33	common_modifications_max	2
34	rare_modifications_max	0
35	%Fixed and variable modifications:	
36	Oxidation / +15.994915 @ F, M, W, Y | common1	
37	Carbamyl / +43.005814 @ NTerm, K, S, T, Y | common1	
38	Carbamidomethyl / +57.021464 @ C | fixed	
39	% Custom modification text below	

40	%Glycan modifications:	
41	Show all N-glycopeptides	0
42	%Addition parameters:	
43	Product Version	PMI-Byonic-Demo:v5.9.5-ga0a5094d

3.10. Workflow for calculation of peptide hydrophobicity factor (HF).

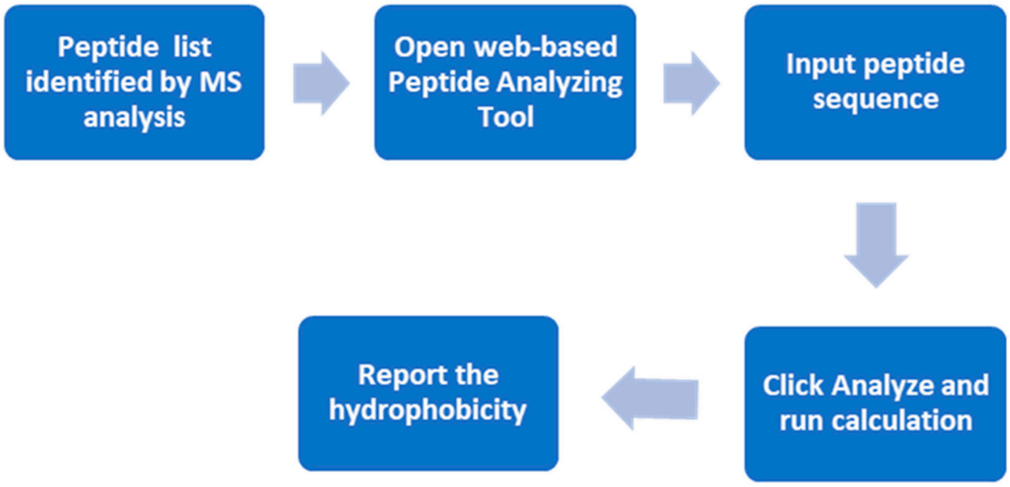

Figure S5. Workflow for calculation of peptide hydrophobicity factor (HF). Peptide sequences identified by LC–MS/MS analysis in this study, as well as peptide sequences from the Pierce™ Peptide Retention Time Calibration Mixture, were entered into the Thermo Fisher web-based peptide analyzing tool and analyzed using default settings. The predicted hydrophobicity value reported by the tool was recorded and defined as the hydrophobicity factor (HF). This procedure was applied consistently to all peptide sequences analyzed in this study.

3.11. The example of calculating HF of peptide TAYLTY

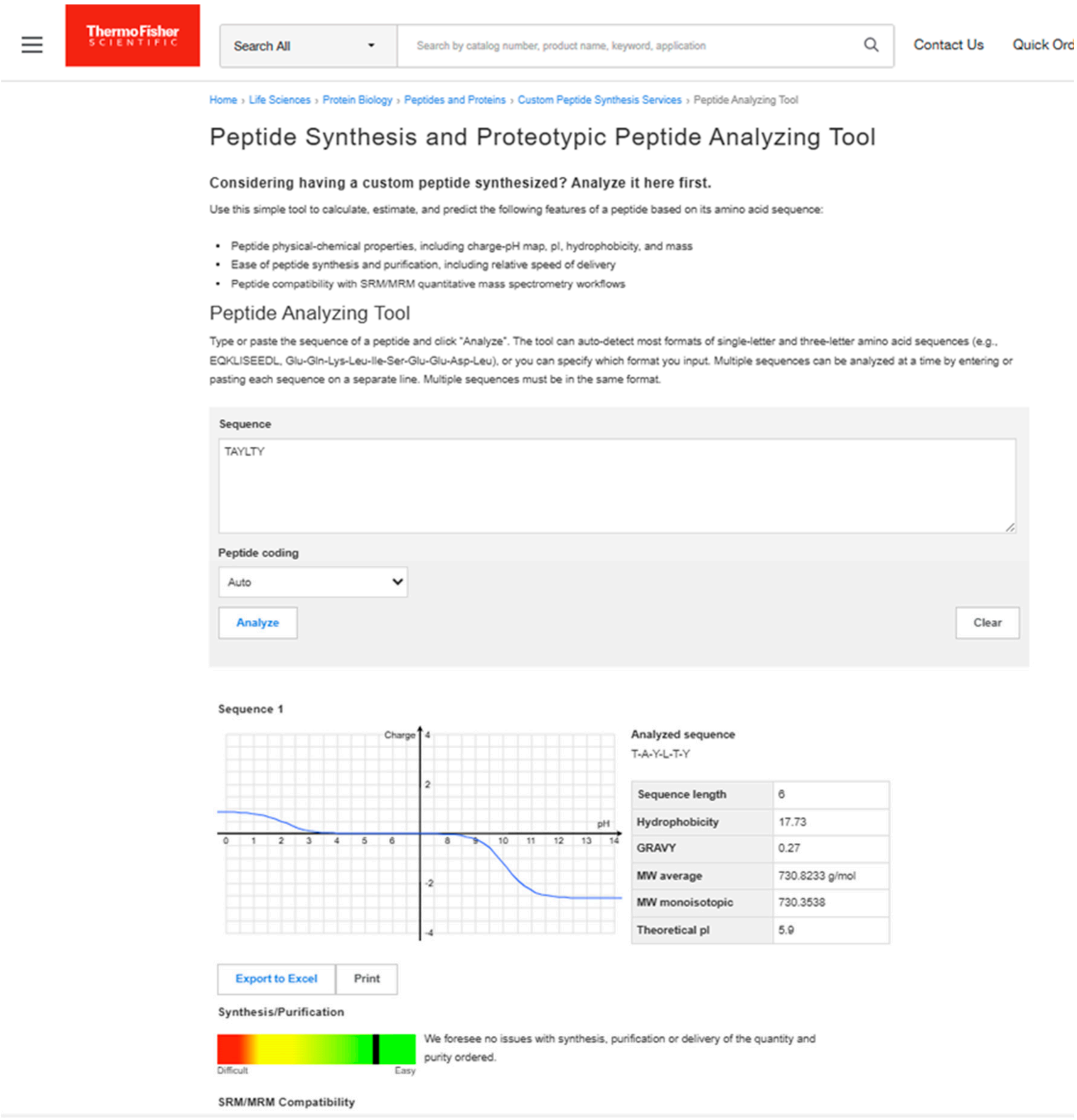

Figure S6. The example of calculating HF of peptide TAYLTY

3.12. Background Protein Analysis

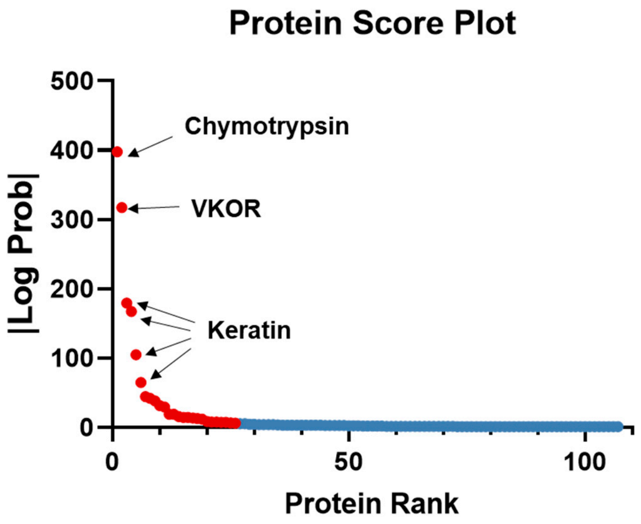

Figure S7. Protein score plot of identified proteins ranked by log p-value. Red points indicate proteins with log p-values at least 2.0 units lower than that of the top decoy protein, while blue points represent all other identified proteins.

Table S7. Proteins identified from the initial database search and used for construction of the focused database.

Protein Rank	Description	|Log Prob|	Best |Log Prob|	Best score	Total Intensity	# of spectra	# of unique peptides
1	>sp|CTRA_BOVIN|(Common contaminant protein)	379.77	10.69	753.4	1.3E+10	716	145
2	>sp|Q2JJF6|VKOR_SYNJB Vitamin K epoxide reductase homolog OS=Synechococcus sp. (strain JA-2-3B'a(2-13)) OX=321332 GN=CYB_2278 P	269.39	7.87	610	2.9E+09	501	135
3	>sp|K2C1_HUMAN|(Common contaminant protein)	122.16	6.29	583.3	1.18E+08	89	55
4	>sp|K1C9_HUMAN|(Common contaminant protein)	84.87	10.54	704.4	87619491	52	34
5	>sp|K1C10_HUMAN|(Common contaminant protein)	70.56	6.11	490.8	46776948	38	26
6	>sp|P0A6Y8|DNAK_ECOLI Chaperone protein DnaK OS=Escherichia coli (strain K12) OX=83333 GN=dnaK PE=1 SV=2	59	6.11	496.5	38581482	34	24
7	>sp|P0AEB2|DACA_ECOLI D-alanyl-D-alanine carboxypeptidase DacA OS=Escherichia coli (strain K12) OX=83333 GN=dacA PE=1 SV=1	53.34	7.12	598.9	50243646	38	26
8	>sp|P37194|SLP_ECOLI Outer membrane protein Slp OS=Escherichia coli (strain K12) OX=83333 GN=slp PE=1 SV=1	49.83	7.47	538.8	71999714	30	14
9	>sp|P0A7A7|PLSB_ECOLI Glycerol-3-phosphate acyltransferase OS=Escherichia coli (strain K12) OX=83333 GN=plsB PE=1 SV=2	43.81	5.24	403.5	5.82E+08	162	57
10	>sp|P0ADB1|OSME_ECOLI Osmotically-inducible putative lipoprotein OsmE OS=Escherichia coli (strain K12) OX=83333 GN=osmE PE=2 SV	36.02	7.39	436.7	11723957	12	11
11	>sp|P0ABJ1|CYOA_ECOLI Cytochrome bo(3) ubiquinol oxidase subunit 2 OS=Escherichia coli (strain K12) OX=83333 GN=cyoA PE=1 SV=1	35.62	7.27	448.8	42069915	37	15
12	>sp|K22E_HUMAN|(Common contaminant protein)	34.25	4.95	573.5	32587250	24	15
13	>sp|P08622|DNAJ_ECOLI Chaperone protein DnaJ OS=Escherichia coli (strain K12) OX=83333 GN=dnaJ PE=1 SV=3	32	6.15	449.9	24747406	20	14
14	>sp|P77775|YFCH_ECOLI Epimerase family protein YfcH OS=Escherichia coli (strain K12) OX=83333 GN=yfcH PE=3 SV=1	27.07	5.99	435.1	17092052	19	11

15	>sp|P0A6J5|DADA_ECOLI D-amino acid dehydrogenase OS=Escherichia coli (strain K12) OX=83333 GN=dadA PE=1 SV=1	17.21	3.71	314.6	9114459	16	13
16	>sp|P00393|NDH_ECOLI Type II NADH:quinone oxidoreductase OS=Escherichia coli (strain K12) OX=83333 GN=ndh PE=1 SV=2	14.44	3.83	296.8	23586592	54	20
17	>sp|P0ACB7|HEMY_ECOLI Protein HemY OS=Escherichia coli (strain K12) OX=83333 GN=hemY PE=1 SV=1	13.19	5.02	433.5	7666676	14	11
18	>sp|P38097|DGCE_ECOLI Probable diguanylate cyclase DgcE OS=Escherichia coli (strain K12) OX=83333 GN=dgcE PE=1 SV=2	13.1	2.32	322.5	84339027	102	36
19	>sp|P0ACB4|HEMG_ECOLI Protoporphyrinogen IX dehydrogenase [quinone] OS=Escherichia coli (strain K12) OX=83333 GN=hemG PE=1 SV=1	12.32	5.07	433.8	5267505	8	6
20	>sp|P0AE14|AMPE_ECOLI Protein AmpE OS=Escherichia coli (strain K12) OX=83333 GN=ampE PE=1 SV=1	12.24	3.8	354	16308464	39	12
21	>sp|P31224|ACRB_ECOLI Multidrug efflux pump subunit AcrB OS=Escherichia coli (strain K12) OX=83333 GN=acrB PE=1 SV=1	11.61	3.84	339.7	14427720	20	13
22	>sp|P17169|GLMS_ECOLI Glutamine--fructose-6-phosphate aminotransferase [isomerizing] OS=Escherichia coli (strain K12) OX=83333	11.48	3.86	393.1	19419297	15	9
23	>sp|P08506|DACC_ECOLI D-alanyl-D-alanine carboxypeptidase DacC OS=Escherichia coli (strain K12) OX=83333 GN=dacC PE=1 SV=2	10.79	3.64	313.4	4889851	7	7
24	>sp|P21177|FADB_ECOLI Fatty acid oxidation complex subunit alpha OS=Escherichia coli (strain K12) OX=83333 GN=fadB PE=1 SV=2	10.42	2.05	238.9	24696869	56	33
25	>sp|P77757|ARNC_ECOLI Undecaprenyl-phosphate 4-deoxy-4-formamido-L-arabinose transferase OS=Escherichia coli (strain K12) OX=83	9.74	4.76	334.8	9475114	9	5
26	>sp|P0AC75|KDTA_ECOLI 3-deoxy-D-manno-octulosonic acid transferase OS=Escherichia coli (strain K12) OX=83333 GN=waaA PE=1 SV=1	8.83	4.27	408.3	15623768	23	14
27	>sp|P30958|MFD_ECOLI Transcription-repair-coupling factor OS=Escherichia coli (strain K12) OX=83333 GN=mfd PE=1 SV=2	7.52	2.2	255.5	1.11E+08	55	30
28	>sp|P31063|YEDD_ECOLI Uncharacterized lipoprotein YedD OS=Escherichia coli (strain K12) OX=83333 GN=yedD PE=1 SV=1	7.47	3.97	271.2	2311489	2	2
29	>sp|P77398|ARNA_ECOLI Bifunctional polymyxin resistance protein ArnA OS=Escherichia coli (strain K12) OX=83333 GN=arnA PE=1 SV=	6.94	4.42	387.3	13684844	21	13

<table>
<tr><td>30</td><td>>sp|P77774|BAMB_ECOLI Outer membrane protein assembly factor BamB OS=Escherichia coli (strain K12) OX=83333 GN=bamB PE=1 SV=1</td><td>6.58</td><td>4.4</td><td>432.9</td><td>29700748</td><td>20</td><td>7</td></tr>
<tr><td>31</td><td>>sp|P68699|ATPL_ECOLI ATP synthase subunit c OS=Escherichia coli (strain K12) OX=83333 GN=atpE PE=1 SV=1</td><td>6.36</td><td>3.35</td><td>263.5</td><td>4441497</td><td>8</td><td>4</td></tr>
</table>
